# Reduction of the Serum Levels of a Specific Biomarker of Cartilage Degradation (Coll2-1) by Hyaluronic Acid (KARTILAGE® CROSS) Compared to Placebo in Painful Knee Osteoarthritis Patients: the EPIKART Study, a Pilot Prospective Comparative Randomized Double Blind Trial

**DOI:** 10.1186/s12891-017-1585-2

**Published:** 2017-05-26

**Authors:** Yves Henrotin, Francis Berenbaum, Xavier Chevalier, Marc Marty, Pascal Richette, François Rannou

**Affiliations:** 1 Bone and Cartilage Research Unit, Arthropole Liège, CHU Sart-Tilman, Liège, Belgium; 2 Rheumatology Department, Sorbonnes Universités UMPC Univ Paris 06, Inserm UMRS 938, DHU i2B, Saint Antoine Hospital, Assistance Publique—Hôpitaux de Paris (AP-HP), Paris, France; 3 Service de Rhumatologie, Hôpital Henri Mondor, Créteil, France; 4 Service de Rhumatologie - Centre Viggo Petersen, Hôpital Lariboisière, Paris, France; 5 University Paris Descartes, PRES Sorbonne Paris Cité, Service de rééducation et réadaptation de l’appareil locomoteur et des pathologies du rachis, Hôpital Cochin, AP - HP, INSERM UMR-S-1124, UFR Biomédicale des Saints Pères, Paris, France

**Keywords:** Viscosupplementation, Treatment, Type II collagen, Metabolism

## Abstract

**Background:**

Viscosupplementation is a symptomatic treatment of the knee osteoarthritis based on the intra-articular injection of hyaluronic acid (IAHA). Although many studies have investigated its effect on symptoms, few clinical studies have focused on its effects on biologicals markers of cartilage metabolism. In this study, we assessed the effect of an intra-articular injection of a reticulated hyaluronic acid compound on the level of a specific biomarker of type II collagen degradation.

**Methods:**

Eighty one patients with symptomatic knee osteoarthritis were included in this randomized placebo controlled trial testing a reticulated hyaluronic acid (HA) with mannitol (KARTILAGE® CROSS, 16 mg/ml, one single injection of 2.2 mL; IAHA) versus saline solution. Primary outcome was the percentage of patients with a reduction of at least 10 nmol/l of serum Coll2-1 between baseline and day 90 (D90, 3 months after injection). Secondary outcomes concerned clinical evaluation and tolerance to the study product.

**Results:**

A significant effect of IAHA was revealed by the sensitivity analysis of the decrease in cartilage marker. In the intention-to-treat population, the percentage of patients showing a decrease in the levels of serum Coll2-1 between inclusion and D90 showed was higher in HA (56.8%) than in placebo group (28.6%; *P* = 0.01). The same significant difference was observed between groups in the per protocol population (57.1% vs 29.0%; *P* = 0.02) corresponding to all patients having received the intra-articular injection (IA), being evaluated for the primary outcome on D-10 and D90, and with no major defined deviation. No significant differences between groups were observed on the changes in function (Lequesne index) or pain and on the number of adverse events.

**Conclusions:**

This is the first randomized double-blind placebo controlled trial showing that IA injection of reticulated HA with mannitol in knee osteoarthritis patients can reduce the serum levels of Coll2-1, a marker specific of type II collagen degradation. This finding suggests that IAHA may have a beneficial effect on cartilage degradation and that Coll2-1 could be used for the assessment of a single intra-articular treatment in clinical trials.

**Trial registration:**

NCT02951585; clinicaltrial.gov. Retrospectively registered on October 28, 2016.

## Background

Osteoarthritis (OA) is one of the most common forms of musculoskeletal disorders and one of the major cause of pain and disability in the adult population [1]. It is a progressive disorder characterized by synovium inflammation, bone remodeling and degradation of the extracellular matrix of articular cartilage [2, 3]. The knee is the most affected joint by OA. It has a high prevalence of 40% for men and 47% for women [4].

Intra-articular (IA) hyaluronic acid (HA) injections, also known as viscosupplementation, are a treatment option for knee OA that serves to restore the decreasing rheological properties of synovial fluid. KARTILAGE® CROSS is a new visco-elastic gel of highly purified reticulated HA. It contains mannitol to provide an anti-oxidative action and to avoid HA depolymerization [5]. Reticulation [6, 7] and mannitol [8–10] increase the residency time of the product in the joint cavity then allowing a single injection in painful knee OA patients.

The US food and drugs administration (FDA) and European medicine agency (EMA), have recently published guidelines recommending a higher level of integration of biomarkers in the development and testing of new drugs to advance decision-making on dosing, time and treatment effect, trial design, and risk/benefit analysis [11]. Biomarkers can be used not only in the process of drug development, but also in the future in assessment of individual patient’s response to treatment as previously described by Kraus et al. [12]. By evaluating the biomarker results, clinicians will be able to conclude whether the treatment has the expected effect or not.

Several soluble biomarkers have been identified as potential candidates to predict or monitor the efficacy of intervention in OA [13]. Among them, type II collagen derivatives have been extensively investigated [14–17]. Coll2-1, a degradation product of type II collagen, has been found in high concentrations in the synovial fluid of human patients with OA compared with healthy controls [18]. This product is generated by the sequential action of collagenase and gelatinase B on type II collagen molecules causing the release of metabolites into synovial fluid [19]. When measured in serum of patients with OA it was found to be in higher concentrations than age-matched controls [18]. Interestingly, Coll2-1 has been shown to be decrease in serum of patients with knee OA after a series of 3 injections of hylan GF-20, suggesting that this biomarker could be helpful for the monitoring of IAHA effect [20]. Coll2-1 is a biomarker of OA that entered the qualification process [21]. Previous studies suggested its potential use for the diagnosis, the prognosis, the burden of disease, and the monitoring of a treatment efficacy. Indeed, it was shown that serum Coll2-1 level increased in OA population by comparison to asymptomatic control group [18]. Moreover, serum Coll2-1 concentration decreased from 6 months after total joint replacement [22]. In urine, the increase of Coll2-1 over 12 months is predictive of radiological OA progression [23]. In preclinical studies, Coll2-1 was qualified as biomarker of burden of disease as the increase of the concentration of the biomarker in serum correlated with the histological severity and the global macroscopic score [24, 25]. Finally, in human clinical trials, Coll2-1 was used to monitor the efficacy of intra-articular viscosupplementation [20] and joint health food supplement [26].

In general, soluble biomarkers are measured in serum and/or in urine. Even if biomarkers level in synovial fluid better reflects what happens in a single joint, synovial fluid collection remains difficult, biomarkers level standardization requires measurement of urea in serum [27], and analytical performance of the assay lower in this fluid due to its rheological properties.

The aim of this study was to evaluate the effects on Coll2-1, a biomarker of cartilage degradation, of an intra-articular injection of hyaluronic acid KARTILAGE® CROSS versus placebo in patients suffering from knee pain. Coll2-1 was measured by an immunoassay that shows very good analytical performances in serum. In addition, clinical efficacy and tolerance for the product were investigated as well as the correlation between Coll2-1 and clinical parameters.

## Methods

### Study design and study population

This study was designed as a 6-month pilot prospective comparative randomized double blind trial testing a new reticulated HA supplemented with mannitol (IAHA) versus saline solution injection in ambulatory knee OA patients.

The study protocol was approved by ethic committee (CPP Ile de France IV) and French national authorities (Agence Nationale de Sécurité du Médicament) (RCB #2012-A01521-42). It was conducted in strict accordance with the declaration of Helsinki and GCP principles. Each patient received and signed an informed consent.

The study was conducted between May 15, 2013 and October 28, 2014.

### Eligibility of patients

Eligible patients were men or women, aged between 45 and 80 years suffering of unilateral symptomatic femoro-tibial knee OA responding to clinical and radiologic ACR (American College of Rheumatology) criteria [28]. OA must have been symptomatic for more than 6 months with a mean global knee pain determined on visual analog scale (VAS) for the last 24 h over 40 mm (without any NSAIDs or analgesics for at least 48 h). Kellgren and Lawrence (K&L) radiological stage must have been II or III. The recruited patients must have required a treatment with hyaluronic acid after failure or intolerance to first line analgesics or NSAIDs. Patients signed their informed consent after receiving comprehensive information.

#### Exclusion criteria

The exclusion criteria were selected to avoid the presence of a contraindication to treatment or diseases affecting biomarkers clearance and to exclude the interference of concomitant painful condition or therapies that may modulate cartilage metabolism. Patients meeting to at least one of the criteria detailed in Table 1 were not included in the study.

**Table 1:** Exclusion criteria

Related to the OA pathology	
○ Radiographical Kellgren and Lawrence grade I or IV ○ Osteoarthritis secondary to a microcrystalline arthropathy: chondrocalcinosis previously known or defined by a calcium border on at least one tibiofemoral spacing, gout …… ○ Isolated femoropatellar OA ○ Presence of another joint (other than the evaluated knee) affected by OA (confirmed in radiographs and symptomatic) ○ Chondromatosis or villonodular synovitis of the knee ○ Paget disease ○ Recent trauma (<1 month) of the evaluated knee ○ Pathologies interfering with the evaluation of OA (radiculalgia in the lower limbs, arteritis…..) ○ Acute inflammatory osteoarthritis (Kofus ≥ 7)	
Related to previous and concomitant treatments	
○ Corticosteroids injection in the evaluated knee in the last month before injection ○ Hyaluronan injection in the evaluated knee in the last 6 months before injection ○ Acetaminophen and NSAIDs 48 h before inclusion visit ○ Change in the dosage of symptomatic slow-acting drugs i.e. chondroitin, glucosamine, diacerein or avocado-soybean unsaponifiables in the last 3 months before first injection ○ Arthroscopy and surgery in the target knee in the last 3 months before first injection ○ Oral corticotherapy	
Related to associated pathologies	
○ Severe diseases (liver or renal failure, uncontrolled cardiovascular diseases….) ○ Other inflammatory rheumatic diseases (e.g. rheumatoid arthritis) ○ Dermatological infection at the site of injection ○ Anticoagulant treatment ○ High risk of hemorrhage	
Related to the patients	
○ Allergy to hyaluronan and constituents (i.e. mannitol) ○ Allergy to acetaminophen ○ Pregnant or breastfeeding women ○ Premenopausal women without contraception ○ Unable to write ○ Participation to a therapeutic clinical trial in the last 3 months ○ Under guardianship or judicial protection	

### Prohibited/authorized treatments

To treat painful condition the following treatments were authorized throughout the study if necessary.

- Acetaminophen (3 g/day) or NSAID at the lowest dose and for the shortest possible period of time.
- Topical NSAIDs
- Non-pharmacologic therapy such as orthosis and physical therapy

These treatments are commonly use in OA clinical trials as rescue treatments for ethical reasons. Number of days of intake of acetaminophen and oral NSAIDs during the month preceding each visit was recorded. However, NSAIDs in suppository or injected through intra-muscular injection, oral corticosteroids, intra-articular injection of corticosteroids, intra-articular injection of hyaluronic acid were forbidden to avoid interaction with outcome results. Slow acting treatments against OA (i.e. chondroitin sulfate, diacerein, avocado soybean unsaponifiable or glucosamine) were allowed throughout the study duration if their dosage was not modified.

### Intervention and randomization

Fifteen combinations of an evaluator physician [general practitioner (GP) or rheumatologist] and an independent injector physician (rheumatologist) of 15 different institutions were involved in the study. Evaluator physician was in charge of patient selection and blinded patient follow up. The randomization list was established using software.

Patients were followed from 10 days before injection (D-10) to 180 days after injection (D180). Inclusion and exclusion criteria were checked at D-10 (inclusion visit) and history of knee OA was recorded. On D0 patients were randomized and were injected (injection visit) into the evaluated knee under aseptic conditions with either Kartilage® Cross, a new reticulated HA supplemented with mannitol (Vivacy, France, 2.2 mL, 16 mg/ml) or saline solution (2.2–2.5 mL, NaCl 9 g/L). The blinded syringes were prepared and provided by the sponsor. Injected products were visually similar.

### Serum preparation and Coll2- 1 immunoassay

Blood collection was done on non-SST dry tubes without heparin. Blood samples were allowed to clot at room temperature for +/− 30 min; the tubes were centrifuged 5–10 min at 2000 rpm at 4 °C. The serum was harvested and stored at −20 °C until assay.

Coll2-1 has been determined in patients’ sera using ELISA kits according to manufacturers’ instructions (Artialis SA, Liège, Belgium). This assay is a competitive immunoassay utilizing a synthetic peptide pre-coated onto the ELISA plate for the quantification of the corresponding antigen in serum samples. A binding competition between the immobilized peptide and the peptide contained in the standards or samples takes place upon addition of the antibody Ab-Coll2-1. After removal of the unbound peptide, a peroxidase-conjugated goat anti-rabbit antibody is added into each well to detect and quantify the level of competitive binding. After washing of the unbound detection antibody, the antibody-antigen complex is detected by a chromogenic reaction with 3,3’,5,5’-tetramethylbenzidine (TMB). The reaction is stopped by adding acid to give a colorimetric endpoint that is subsequently determined spectrophotometrically. The limits of quantification for the assay ranged from 31.25 nM to 2000 nM.

### Outcome measures

#### Primary outcome

The main objective of this study was to evaluate the effect of IAHA on cartilage metabolism. In this purpose, we selected the most cartilage specific sequence Coll2-1. This type II collagen sequence is released during cartilage degradation by metalloproteases and directly measured in the serum using an immunoassay. The primary outcome was the percentage of patients with a reduction of at least 10 nmol/l of serum Coll2-1 between visit (D-10) and D90 (3 months after injection). The cut-off value of 10 nmol/l was based on the measurement error of the serum Coll2-1 immunoassay. The smallest detectable difference (SDD) of the serum Coll2-1 immunoassay was evaluated by the method of Bland and Altman [29] from 22 sera measured two times at two different days.

#### Secondary outcomes

The secondary outcomes (Table 2) included the change in Coll2-1 levels between inclusion (D-10), D30, and D180 in order to document changes in Coll2-1 levels throughout the study. Additionally, changes in pain and function as well as the use of rescue medication were considered.

**Table 2:** Secondary outcomes

Parameter	Method of analysis
Coll2-1	Decrease between inclusion (D-10) and D30 or D180
Lequesne index (LI)	Decrease between inclusion visit (D-10) and further visits
Global assessment of pain	Visual analog scale (VAS, 0–100 mm) between inclusion visit (D-10) and further visits
OMERACT/OARSI set of criteria	Percentage of responders at D90 and D180
Acetaminophen and NSAID consumption	Number of patients that required at least once rescue medication during the study
Patient’s global assessment of the disease activity	11 points Likert scale ranging of 0 (low active disease) to 10 (maximum activity)

*Abbreviations*: *OMERACT* Outcome Measures in Rheumatology, *OARSI* Osteoarthritis Research Society International, *D-10* 10 days before injection, *D30, D90, D180* respectively 30, 90, and 180 days following injection

#### Tolerance

Tolerance was assessed through the monitoring of adverse events (AEs). Each one was detailed. The patients’ global tolerance to the study product was also recorded (scale 0–5, ranging from 0: excellent to 5: very bad).

### Statistical analysis

#### Populations

Several populations were defined.

The population of included patients corresponded to the patients who were selected and included in the study on D-10.

The intent to treat (ITT) population corresponded to the included and randomized patients.

The tolerance population comprised all randomized patients, having received the intra-articular injection with the study product.

The full analysis set (FAS) population represented all randomized patients, having received the intra-articular injection of the study product, being evaluated for the primary outcome on D-10 and at least once after the injection of the study product. Missing values were handled with the LOCF (last observed carried forward) technique. This means that D-10 data should be available and that if D90 data was missing, the result of the closest measurement was taken into account.

Per protocol (PP) population corresponded to all patients of the FAS population who did not present any major defined deviation. The protocol defined as major the following deviations:

- Those related to inclusion or exclusion criteria
- Missing of the primary outcome on D90
- Intake of a treatment that was forbidden and that could have an important impact on the primary outcome
- Any deviation that could have a major impact on the primary outcome.

#### Sample size

HA injections in a previous study including 45 patients produced a decrease in Coll2-1 superior to 10 nmol/l in 48% of the patient [20]. The hypothesis was made that Kartilage® Cross treatment in this study will produce a decrease of 10 nmol/l of Coll2-1 between inclusion and D90 in the serum in 45% of the patients. Hence 35 patients per group would produce a difference of 33% with placebo group (12% expected in the placebo group) considering a risk of 5% and a power of 80% (Fleiss method with continuity correction). Taking into account patients who would be out of the study, the number of patients per group was defined as 40.

#### Analysis of the primary outcome

The analysis of the efficacy primary outcome (% of patients with a reduction of at least 10 nmol/l of serum Coll2-1 between D-10 and D90) was performed on the FAS population using the LOCF. A Chi-square test or a Fisher Exact was used. Sensitivity analysis were done on FAS population without handling of missing data (analysis on observed cases) and on PP population.

#### Analysis of the secondary outcomes

The change in both clinical and biological parameters was analyzed if the distribution of the population was normal, using an ANCOVA with an adjustment on the basal value. In case of non-normal distribution, a non-parametric test was used. Analyses were conducted on the FAS population. LOCF approach was considered to deal with missing data. Data were analyzed at each time point versus baseline (between D-10/D30, D-10/D90, and D-10/D180) and were completed by an ANOVA for repeated measures based on the time D-10/D30/D90/D180 and the treatment if applicable.

Response to the test product according to OMERACT/OARSI criteria were analyzed using a Chi-square test at D30, D90 and D180 on the FAS population dealing with missing data (VAS and LI) using the LOCF approach. The consumption of acetaminophen and NSAIDs were compared between groups with a Chi-square test or Fisher Exact test on the FAS population without considering missing data on D30, D90 and D180.

#### Assessment of safety

The percentage of patient with at least one AE, one serious AE, one AE attributable to the intervention, one AE attributable to the product, and one event leading to study stopping were recorded. The percentage of the most frequent local or systemic events were calculated and compared using a Chi-square test or Fisher Exact test depending on their occurrence. They were presented with regards to their link to the product, their evolution and the therapeutic decision. The serious intercurrent events were described and compared.

The global tolerance appreciated by the patient was analyzed using a non-parametric test on rank values.

## Results

### Study populations

Eighty four (84) patients were selected. Three of them did not attend the injection visit and were not randomized in the study. Eighty one (81) patients were randomized in the study: 40 in the treatment group and 41 in the saline solution group. Four patients finished prematurely the study: one (2.5%) from the treatment group due to AE, 3 (7.3%) from the saline solution group (one for AE, one for AE and inefficacy, one for loss of follow up at D180). Five patients in the treatment group (17%) and 10 (30%) in the saline solution group presented a major deviation to protocol. Major deviations were related to the non-respect of delays between visits. The ITT and FAS populations contained 81 patients (40 in the treatment group and 41 in the saline solution group) and the PP population contained 66 patients (35 in the treatment group and 31 in the saline solution group).

### Patient characteristics

Both groups of the study were not statistically different for their demographic and morphological criteria (Table 3). Patients were mainly women (69.1%) with a mean age of 65.0 ± 9.8 years and a mean body mass index (BMI) of 29.9 ± 7.3 kg/m^2^. Patients were suffering from OA for 6.7 ± 6.8 years. 55.6% (*n* = 45) had a K&L of II and 44.4% (*n* = 36) of III and 54.3% (*n* = 44) had pain during night. 49.4% of patients had not taken any treatment for OA for the past 3 months. 32.1, 13.6 and 8.6% had taken analgesics, NSAIDs and/or slow acting anti OA drugs respectively. No significant difference was reported between groups for either parameter.

**Table 3:** Demographic data and OA history of the FAS population (*N* = 81)

	IAHA *N* = 40	Saline solution *N* = 41	Total *N* = 81	*P* value	Test
Age (years)	66.9 ± 10.4	63.0 ± 8.9	65.0 ± 9.8	0.0752	Student
Sex					
- *Women* - *Men*	62.5% 37.5%	75.6% 24.4%	69.1% 30.9%	0.2016	Chi-square test
BMI (kg/m^2^)	29.0 ± 7.4	30.8 ± 7.2	29.9 ± 7.3	0.2465	Student
Disease duration (year)	7.6 ± 8.0	5.9 ± 5.3	6.7 ± 6.8	0.2639	Student
Evaluated knee					
- Right	45.0%	48.8%	46.9%		
- Left	55.0%	51.2%	53.1%	0.7332	Chi-square test
Radiological score (K&L)					
- Score II	52.5%	58.5%	55.6%		
- Score III	47.5%	41.5%	44.4%	0.5846	Chi-square test
Night pain	52.5%	56.1%	54.3%	0.7452	Chi-square test
Effusion	20.0%	14.6%	17.3%	0.5231	Chi-square test
OA treatment for the past 3 months					
- Acetomiphen	27.5%	41.5%	32.1%	0.2155	Chi-square test
- NSAIDS	15.0%	12.2%	13.6%	0.7126	Chi-square test
- slow acting anti OA drugs	7.5%	9.8%	8.6%	0.7179	Chi-square test

### Primary efficacy outcome

The percentage of patient with a reduction of at least 10 nmol/l of serum Coll2-1 between D-10 and D90 was 52.5% in the treatment group and 31.7% in the placebo group in the FAS population when dealing with the missing values. This difference was not statistically significant (*P* = 0.0580).

The sensitivity analysis of the primary outcome revealed a significant difference between the two groups in the FAS population without handling the missing values. 56.8% of the patients in the treatment group had a reduction of at least 10 nmol/l of serum Coll2-1 between D-10 and D90 whereas 28.6% in the placebo group did (*P* = 0.0158) (Table 4). The same statistically significant difference was observed in the PP population without considering the missing data (Table 5).

**Table 4:** Variation of serum Coll2-1 between D-10 and D90 in the FAS population without handling the missing values

	IAHA *N* = 40 at D-10 *N* = 37 at D90	Saline solution *N* = 41 at D-10 *N* = 35 at D90	*P* value	Test
Serum Coll2-1 at D-10	840.3 ± 375.8 (*N* = 40)	766.1 ± 359.2 (*N* = 41)	0.3663	ANOVA
Serum Coll2-1 at D90	745.4 ± 343.5 (*N* = 37)	782.3 ± 233.7 (*N* = 35)	0.5975	ANOVA
Adjustment on basal value	−80.2 ± 44.1	−14.6 ± 45.3	0.3053 0.0030**	ANCOVA Wilcoxon^a^
Reduction of at least 10 nmol/l	56.8%	28.6%	0.0158*	Chi-square test

^a^Shapiro-Wilk normality test < 0.0001, basal value was used as covariate

**p < 0.01, *p < 0.05

**Table 5:** Variation of serum Coll2-1 between D-10 and D90 in the PP population

	IAHA *N* = 35	Saline solution *N* = 31	*P* value	Test
Serum Coll2-1 at D-10	859.2 ± 396.6	763.7 ± 389.8	0.3288	ANOVA
Serum Coll2-1 at D90	745.9 ± 353.1	767.4 ± 204.0	0.7674	ANOVA
Adjustment on basal value	−82.2 ± 45.6	−31.5 ± 48.5	0.4500 0.0057**	ANCOVA Wilcoxon^a^
Reduction of at least 10 nmol/l	57.1%	29.0%	0.0217*	Chi-square test

^a^Shapiro-Wilk normality test < 0.0001, basal value was used as covariate

**p < 0.01, *p < 0.05﻿

**Fig. 1:**
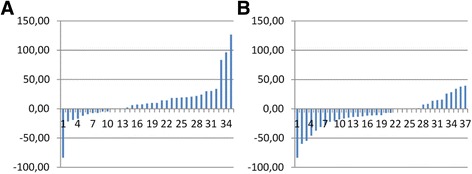
Individual change of Coll2-1 level in serum of patient receiving placebo (**a**) or treated with Kartilage Cross (**b**)

The percentage changes in each patient's biomarker levels from baseline to D90 (primary outcome) was calculated and displayed on Fig. 1.

The number of patients showing a decrease in Coll2-1 was higher in the Kartilage Cross (22/37) group than, in the placebo group (10/34). Inversely, more patients showed an increase of Coll2-1 in the placebo (20/34) than in the Kartilage cross group (9/37).

### Secondary efficacy outcomes

#### Biological efficacy

The biological efficacy of the treatment was evaluated using the variation in Coll2-1 between inclusion (D-10) and D30 or D180 as secondary outcome.

No statistically significant difference was observed regarding the change in Coll2-1 from inclusion to D30. However, change in Coll2-1 between D-10 and D180 showed statistically significant difference between groups (−34.5 ± 49.7 nmol/l for IAHA vs 43.5 ± 49.0 nmol/l for placebo, *P* = 0.0473, Table 6).

**Table 6:** Variation of serum Coll2-1 between D-10 and D180 in the FAS population without handling the missing values

	IAHA *N* = 40 at D-10 *N* = 35 at D180	Saline solution *N* = 41 at D-10 *N* = 36 at D180	*P* value	Test
Serum Coll2-1 at D-10	840.3 ± 375.8 (*N* = 40)	766.1 ± 359.2 (*N* = 41)	0.3663	ANOVA
Serum Coll2-1 at D180	784.0 ± 223.2 (*N* = 37)	865.1 ± 343.9 (*N* = 36)	0.244	ANOVA
Adjustment on basal value	−34.5 ± 49.7	43.5 ± 49.0	0.2689 0.0473*	ANCOVA Wilcoxon^a^

^a^Shapiro-Wilk normality test < 0.0001, basal value was used as covariate﻿

*p < 0.05﻿

#### Clinical efficacy (Table 7)

No statistically significant difference was observed between groups regarding the change in function (LI) or pain (VAS). There was also no difference regarding the OMERACT-OARSI responders and the patient global assessment on disease activity.

**Table 7:** Change in clinical secondary outcome on the FAS population (LOCF approach)

		D-10	D30	D90	D180	Test
LI	Treatment group (*N* = 40)	12.5 ± 3.8	8.3 ± 4.1	8.2 ± 4.3	6.8 ± 4.9	ANOVA
	Placebo group (*N* = 41)	12.5 ± 3.4	9.0 ± 5.0	8.9 ± 5.5	8.1 ± 5.5	
	*P* value	0.9500	0.4983	0.5466	0.3395	
Change in pain (VAS)	Treatment group (*N* = 40)	65.7 ± 11.6	35.9 ± 21.5	31.4 ± 24.2	27.9 ± 23.2	ANOVA
	Placebo group (*N* = 41)	66.4 ± 9.8	38.6 ± 21.6	36.2 ± 25.6	30.8 ± 23.9	
	*P* value	0.7719	0.5659	0.3939	0.5738	
Change in pain intensity adjusted to basal value (VAS)	Treatment group (*N* = 40)	–	−30.1 ± 3.4	−34.8 ± 3.9	−38.2 ± 3.7	ANOVA
	Placebo group (*N* = 41)	–	−27.5 ± 3.4	−29.7 ± 3.8	−35.2 ± 3.7	
	*P* value	–	0.5925	0.3543	0.5576	
Change in pain intensity responder 30% (VAS)	Treatment group (*N* = 40)	–	60.0%	70.0%	72.5%	Chi-square test
	Placebo group (*N* = 41)	–	61.0%	65.9%	75.6%	
	*P* value	–	0.9284	0.6894	0.7495	
Change in pain intensity responder 50% (VAS)	Treatment group (*N* = 40)	–	50.0%	50.0%	57.5%	Chi-square test
	Placebo group (*N* = 41)	–	43.9%	43.9%	63.4%	
	*P* value	–	0.5825	0.5825	0.5862	
%OMERACT -OARSI responders/non-responders	Treatment group (*N* = 40)	–	70.0%	70.0%	67.5%	Chi-square test
	Placebo group (*N* = 41)	–	70.7%	65.9%	61.0%	
	*P* value	–	0.9425	0.1597	0.3750	
Patient global assessment on disease activity	Treatment group (*N* = 40)	6.6 ± 1.3	3.6 ± 1.9	3.4 ± 2.5	2.9 ± 2.1	ANOVA
	Placebo group (*N* = 41)	6.4 ± 1.3	4.0 ± 2.1	4.1 ± 2.5	3.5 ± 2.4	
	*P* value	0.6411	0.447	0.2052	0.2444	

##### Rescue medication

No significant difference (FAS population) was observed between the 2 groups regarding the consumption of acetaminophen (50.0% vs 53.7% between D0 and D30, 55.0% vs 52.5% between D30 and D90 and 35.0% vs 47.4% between D90 and D180 for IAHA and saline solution respectively) and or NSAIDs (7.5% vs 7.3% between D0 and D30, 7.5% vs 7.5% between D30 and D90 and 10.0% vs 2.6% between D90 and D180 for IAHA and saline solution respectively).

##### Tolerance

Thirty AEs were reported in 29 patients among 81 (35.8%) during the study, 14 in the IAHA group and 15 in the saline solution group. Two AEs were considered attributable to the product, one joint effusion in the treatment group and one inflammation at the injection site in the saline solution group. Three AE were considered as serious, one in the treatment group and 2 in the placebo group. None of them was considered as related to the product or the procedure of injection.

## Discussion

This is the first clinical trial designed in order to study the effect of IAHA in knee OA patients on serum levels of Coll2-1, a biomarker OA of cartilage degradation. Despite the absence of significant effect on the primary end-point, this study shows that a single intra-articular injection of a new reticulated HA supplemented with mannitol may reduce serum levels of Coll2-1. Coll2-1 is a degradation product of type II collagen released during cartilage degradation.

This study is confirmatory of the BIOVISCO study demonstrating that three injections of hylan G20 decreased serum Coll2-1 [20]. This also confirms the sensitivity of this biomarker to metabolic change occurring in a single joint. Another key observation was the absence of relationship between Coll2-1 variation and the clinical response, probably due to the absence of effects of the product on symptoms.

This study is an additional step in the qualification of Coll2-1. A biomarker is defined as a characteristic that is objectively measured and evaluated as an indicator of normal biologic processes, pathogenic processes, or pharmacologic responses to a therapeutic intervention. Among other things, biomarkers in the OA field can be used in drug development, treatment monitoring and the future basis of personalized evidence-based action plans. According the BIPED classification, an efficacy of intervention biomarker has to be indicative or predictive of treatment efficacy. The concentration of this biomarker has to differ significantly between patient populations with or without treatment, or before and after within patient. Based on our results, Coll2-1 could be considered as a valuable tool for monitoring IAHA effect at an individual level. Of course, large cohort and meta-analysis performed on some clinical study should be conducted to complete the qualification process of this marker.

An intriguing result is the absence of effects of IAHA on the clinical parameters, while some previous studies have reported a beneficial effect [30, 31]. The absence of clinical effect may be explained by the design of the study built to study the effects of HA on Coll2-1. For example, the sample size was estimated to detect an effect on Coll2-1, but not to detect an effect on algo-functional status. Another explanation may be the lack of correlation between the degradative process illustrated by the serum levels of biomarkers and the clinical evolution. Moreover, the population is composed in majority by women with unilateral knee OA, two factors of prognosis of a lesser clinical responses. The criteria chosen in this study for clinical evaluation were the one classically used and previously defined by Altman et al. [28]. In addition the placebo effect was clearly described in OA patients, especially after an invasive intervention as IA injection [32]. This is then paramount to design clinical trial that will not only prove the clinical efficacy of a treatment but also the relevance of biomarkers.

This study has several limitations. One the major limitation is that the effect is significant on the FAS population if the missing value were not handled, but not if this missing value were replaced by the LOCF. A second concern was the absence of clinical efficacy of the product. This limits the possibility to correlate biomarker levels with clinical outcomes and then to qualify the biomarker as a marker of efficacy. Moreover it was designed as a pilot study and even though it allowed obtaining significant results in a small number of patients. Finally, our biomarker assessment is limited to Coll2-1, a biomarker of type II collagen degradation. The measurement of a biomarker evaluating type II collagen synthesis would have been helpful to better appreciate the effect of IAHA on cartilage metabolism. Further investigations would confirm and detail the effect of this reticulated HA formulation.

## Conclusion

For the first time, this study demonstrated that IAHA decreased significantly serum Coll2-1, a maker of cartilage catabolism, compared to the injection of a saline solution. This finding suggests that IAHA may have a beneficial on cartilage degradation and suggests that Coll2-1 could be used for the assessment of a single intra-articular treatment in clinical trials.

## Abbreviations

ACR: American college of rheumatology
AE: Adverse event
D: Day
EMA: European medicine agency
FAS: Full Analysis Set
FDA: Fodd and drug administration
GP: General practitioner
HA: Hyaluronic acid
IA: Intra-articular
ITT: Intent To Treat
K&L: Kellgren and Lawrence
LI: Lequesne index
LOCF: Last observation carried forward
NSAID: Non-steroidal anti-inflammatory drug
OA: Osteoarthritis
OARSI: Osteoarthritis research society international
OMERACT: Outcome Measures in Rheumatoid Arthritis Clinical Trials
PP: Per propocol
VAS: Visual analog scale
